# Exploratory Use of Decision Tree Analysis in Classification of Outcome in Hypoxic–Ischemic Brain Injury

**DOI:** 10.3389/fneur.2018.00126

**Published:** 2018-03-06

**Authors:** Thanh G. Phan, Jian Chen, Shaloo Singhal, Henry Ma, Benjamin B. Clissold, John Ly, Richard Beare

**Affiliations:** ^1^Stroke and Aging Research Group, School of Clinical Sciences, Department of Medicine, Monash University and Stroke Unit, Monash Medical Centre, Melbourne, VIC, Australia; ^2^Murdoch Children’s Research Institute, Melbourne, VIC, Australia

**Keywords:** cardiac arrest, hypoxic ischemic encephalopathy, decision tree analysis, classification, prediction

## Abstract

**Background:**

Prognostication following hypoxic ischemic encephalopathy (brain injury) is important for clinical management. The aim of this exploratory study is to use a decision tree model to find clinical and MRI associates of severe disability and death in this condition. We evaluate clinical model and then the added value of MRI data.

**Method:**

The inclusion criteria were as follows: age ≥17 years, cardio-respiratory arrest, and coma on admission (2003–2011). Decision tree analysis was used to find clinical [Glasgow Coma Score (GCS), features about cardiac arrest, therapeutic hypothermia, age, and sex] and MRI (infarct volume) associates of severe disability and death. We used the area under the ROC (auROC) to determine accuracy of model. There were 41 (63.7% males) patients having MRI imaging with the average age 51.5 ± 18.9 years old. The decision trees showed that infarct volume and age were important factors for discrimination between mild to moderate disability and severe disability and death at day 0 and day 2. The auROC for this model was 0.94 (95% CI 0.82–1.00). At day 7, GCS value was the only predictor; the auROC was 0.96 (95% CI 0.86–1.00).

**Conclusion:**

Our findings provide proof of concept for further exploration of the role of MR imaging and decision tree analysis in the early prognostication of hypoxic ischemic brain injury.

## Introduction

Hypoxic coma carries the highest mortality rate among the different causes of coma with only around 30% of patients admitted to ICU ever regaining awareness (1). There is increasing interest in this condition given the changing landscape of hypoxic ischemic brain injury in the context of therapeutic hypothermia (2–4). Many patients require ongoing life support after resuscitation, and the decision to continue treatment is heavily influenced by the likely prognosis. The ability to accurately predict long-term outcome is therefore important for a balanced approach to decision making and aid allocation of health-care resources to optimize individual outcomes. A recent meta-analysis from our group reported that the clinical examination at day 2 was the best predictor of outcome following coma above that provided by sensory evoked potential and electroencephalography (5). That analysis did not include imaging data. There are very few publications in the literature that address the role of MR imaging in the prediction of coma outcome (6, 7).

Most models predicting outcome of hypoxic ischemic coma have been developed using regression methodology. We are aware of one model (incorporating electrophysiological variables rather than MR imaging) developed using classification and regression tree analysis (CART) (8). Decision tree methods generate a logical flow chart diagram which resembles a tree (9). This tree like diagram, with repeated partitioning of the original data into smaller groups (nodes) on a yes or no basis, mimics clinical pathway reasoning. In this exploratory analysis, we evaluated the potential of MR imaging to improve outcome prediction in the era of therapeutic hypothermia in cardiac arrest patients, using the framework of decision tree analysis.

## Materials and Methods

The data here have been discussed in our previous study, which compared methodologies for comparison of topographic imaging findings, rather than outcome prediction (10).

### Patient Selection

Patients who presented to Monash Health between December 2003 and January 2011 with cardiac arrest were included. The inclusion criteria were: age ≥17 years, a clinical diagnosis of coma on ICU admission, and no contraindications to MR Imaging. We extracted demographic data (age and sex) and data related to coma such as Glasgow Coma Score (GCS), whether the event was witnessed and the down time. The down time was defined as the time that the person became unconscious with no cardiac output to the time at which the cardiac output returned. In this study, disability was defined according to the modified Rankin Scale (mRS). Mild disability was defined as mRS at 90 days of 0–2, moderate as mRS 3–4, and severe disability and death as mRS 5–6. This project was approved by the Monash Health Ethics committee.

### Imaging Techniques

MRI scans were performed on 1.5 T superconducting imaging systems (General Electric Medical Systems, Milwaukee, WI, USA and Siemens Medical Solutions, Malvern, PA, USA) with echo-planar imaging capabilities. Fluid attenuated inversion recovery (FLAIR) (TR = 8,802 ms, TE = 130 ms, TI = 2,200 ms, voxel size 0.50 mm × 0.50 mm × 5 mm). Diffusion-weighted imaging (DWI) was performed using EPI techniques with 6/1.7 mm thickness, matrix 128 × 256, field of view 230 mm, and TR/TE 10,000/102 ms. Diffusion gradient values (*b* values) of 0 and 1,000 s/mm^2^ were applied in three directions. The images with the 1,000 s/mm^2^ diffusion gradient are referred to here as DWI images. Apparent diffusion coefficient (ADC) maps were calculated using the Stesjkal and Tanner equation on a voxel by voxel basis.

### Segmentation and Registration

The imaging method was described in a previous paper from our group (10). In brief, infarct tissue was manually segmented on T_2_-weighted and B1000 images of the DWI sequence. On the ADC map, voxels were empirically defined as abnormal if the ADC values were ≤800 × 10^−6^ mm/s^2^. This empirical definition would result in more voxels being classified as infarcted compared with a value lower than 800 × 10^−6^ mm/s^2^. The infarct volume was calculated by finding the union of infarct tissue (total infarct volume) on the FLAIR, B1000 image, and ADC images.

### Statistical Analysis

In this study, we used decision tree analysis to develop models for discriminating between mild disability and severe disability or death. The tree construction was performed using a free version of CART known as *rpart* (available from R Foundation, http://cran.r-project.org/web/packages/rpart/rpart.pdf). The classification can be viewed as a set of rules that are applied sequentially with each rule partitioning an attribute (predictor variable) into a binary response. The method uses a splitting rule built around the notion of “purity.” A node in the tree is defined as pure when all the elements belong to one class. When there is impurity in the node, a split occurs to maximize reduction in “impurity.” In some case, the split may be biased toward attributes that contain many different ordinal levels or scales (11). Thus the selection of an attribute as the *root* node may vary according to the splitting rule and the scaling of the attribute (11). We refer to this type of analysis as a binary decision tree to indicate partitioning of the data into two groups. One major advantage of *rpart* is the presentation of the classification rules in the easily interpretable form of a tree. The hierarchical nature of the decision tree is similar to many decision processes. The data were partitioned into training (4/5) and validation (1/5). The accuracy of the model was assessed using the area under the ROC (auROC) and interpreted using the guidelines set by Hosmer and Lemeshow (12). An auROC of 0.5 is classified as no better than by chance; 0.6–0.69 provides poor discrimination; 0.7–0.79 provides acceptable (fair) discrimination; 0.8–0.89 provides good (excellent) discrimination, and 0.9–1.0 provides outstanding discrimination (12).

#### Model 1a: Imaging Data at Day 0

The decision tree was grown using the following predictors: *infarct volume*, GCS at day 0, witnessed cardiac arrest, estimated duration of downtime to cardiopulmonary resuscitation, cooling, age, and sex. For the purpose of these exploratory analyses, we make the assumption that the infarct volume remains constant from day 0 to day 7.

#### Model 1b: No Imaging Group at Day 0

The decision tree was grown using the following predictors: GCS at day 0, witnessed cardiac arrest, estimated duration of downtime to cardiopulmonary resuscitation, cooling, age, and sex.

#### Model 2a: Imaging Data at Day 2

The decision tree was grown using the following predictors: *infarct volume*, GCS at day 2, witnessed cardiac arrest, estimated duration of downtime to cardiopulmonary resuscitation, cooling, age, and sex.

#### Model 2b: No Imaging Group at Day 2

The decision tree was grown using the following predictors: GCS at day 2, witnessed cardiac arrest, estimated duration of downtime to cardiopulmonary resuscitation, cooling, age, and sex.

#### Model 3a: Imaging Data at Day 7

The decision tree was grown using the following predictors: infarct volume, GCS at day 7, witnessed cardiac arrest, estimated duration of downtime to cardiopulmonary resuscitation, cooling, age, and sex.

#### Model 3b: No Imaging Data at Day 7

The decision tree was grown using the following predictors: GCS at day 7, witnessed cardiac arrest, estimated duration of downtime to cardiopulmonary resuscitation, cooling, age, and sex.

## Results

### General Characteristics

A total of 309 patients with cardiac arrest presented to Monash Medical Centre between December 2003 and January 2011. Forty-one (13.3%) patients met the inclusion criteria (coma following cardiac arrest, ICU admission, and MR imaging) and were included in our study. The mean age ± SD was 51.5 ± 18.9 years. Fifty-nine percent of the studied patients received cooling therapy. The mean down time was 24.3 ± 14.0 min. The median time to MR imaging among the group with mRS 6 was 4.5 days (interquartile range 3.3 and 13.0 days). The median time to MR imaging for the remainder (mRS 0–5) was 10.0 days (interquartile range 6.3 and 13.0 days). The median time from MR imaging to death was 6 days (interquartile range 3.3 and 15 days). Approximately 27.8% of patients died 3 days after MR imaging.

### Decision Tree Models

**Figure 1:**
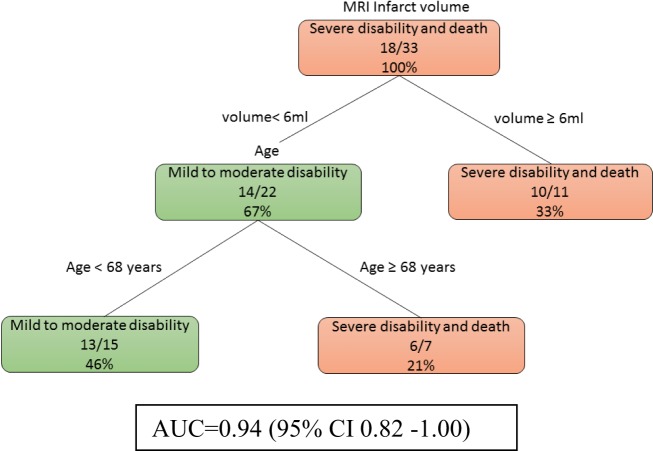
The decision tree model incorporating infarct volume at day 0 and day 2. The poor outcomes are labeled in orange boxes, and the good outcomes are labeled as green boxes. Age is important when the infarct volume is less than 6 ml. The area under receiver operating curve for training data was 0.94 (95% CI 0.82–1.00).

At day 0, model 1a (Figure 1) showed that age is important when the infarct volume is less than 6 ml. Patients with larger infarct volume (>6 ml) did poorly regardless of their age. By contrast, younger patients (<68 years old) with small infarct volume (<6 ml) had mild to moderate disability outcome. The training data consists of four-fifths of 41 patients or 33 patients. The auROC for training data was 0.94 (95% CI 0.82–1.00). The validation data consist the remaining one-fifth of 41 patients or 8 patients. The auROC for validation data was 0.85 (95% CI 0.45–1.00).

**Figure 2:**
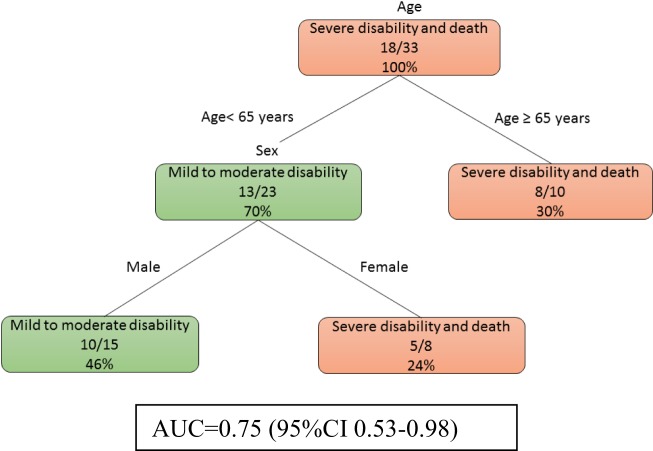
The decision tree model for patients without MRI at day 0. The poor outcomes are labeled in orange boxes, and the good outcomes are labeled as green boxes. Age was the main determinant of poor outcome. The area under receiver operating curve for training data was 0.75 (95% CI 0.53–0.98).

At day 0, model 1b (Figure 2) showed that age was the main determinant of poor outcome followed by female patients (imaging data not used here). Among the male patients, those of younger age (<65 years) were less likely to have severe disability. The auROC for training data was 0.75 (95% CI 0.53–0.98). The auROC for validation data was 0.85 (95% CI 0.45–1.00).

At day 2, model 2a was identical to model 1a in that age and infarct volume were important predictors for poor outcome prediction at day 2.

**Figure 3:**
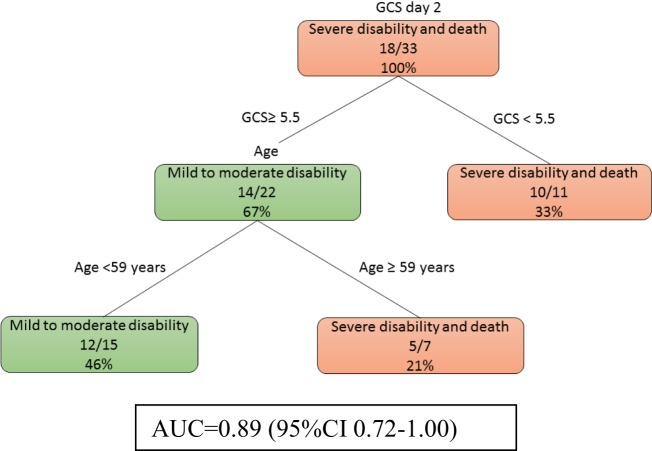
The decision tree model without MRI at day 2. The poor outcomes are labeled in orange boxes, and the good outcomes are labeled as green boxes. Patients with low Glasgow Coma Score (GCS) (<5.5) had poor outcome while older patients do poorly even if their GCS was higher. The area under receiver operating curve for training data was 0.89 (95% CI 0.72–1.00).

At day 2, model 2b (Figure 3) showed that a low GCS predicted poor outcome (imaging data not used here). The prognosis of those patients with higher GCS (≥5.5) can be further defined by their age. The auROC for training data was 0.89 (95% CI 0.72–1.00). The auROC for validation data was 0.85 (0.45–1.00).

**Figure 4:**
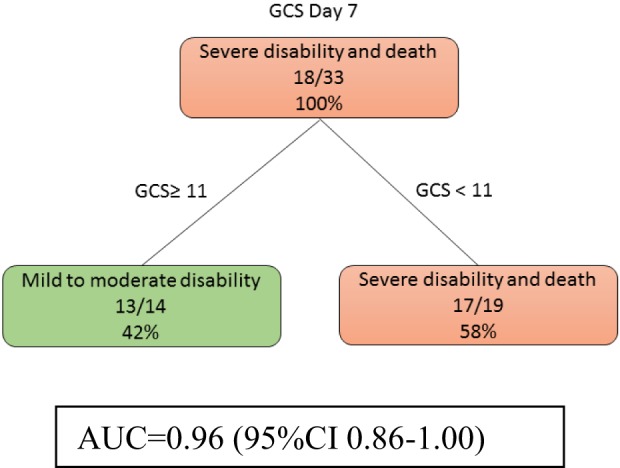
The decision tree model at day 7 shows that Glasgow Coma Score (GCS) discriminate disability outcome. The poor outcomes are labeled in orange boxes, and the good outcomes are labeled as green boxes. Infarct volume did not help to classify good from poor outcome at this stage. The area under receiver operating curve for training data was 0.96 (95% CI 0.86–1.00).

At day 7, models 3a and 3b were identical. Patients with low GCS (<11) were likely to have severe disability (Figure 4). The auROC for training data was 0.96 (95% CI 0.86–1.00). The auROC for validation data was 0.95 (95% CI 0.65–1.00).

## Discussion

In this study, we used binary decision trees to explore associations between clinical variables and severe disability following hypoxic ischemic brain injury. The models were developed to explore their potential application by clinicians at different stages of illness. For this reason, we have developed the models with and without incorporation of infarct volume. These approaches have resulted in decision tree models with outstanding discrimination for clinical outcome, even in the early stage of the intensive care management. However, there are caveats—these models were created from data of small sample size, and there were strong assumptions about the lack of changes in ischemic volume on MR scans. As such, we caution the readers not to use the findings for clinical purpose and to consider the findings here as proof of concept.

Our choice of decision tree analysis was based on its similarity to clinical pathway and ease of understanding by clinicians. The decision tree package *rpart* does tolerate certain degree of missing number because the data are split using the available data for that attribute to calculate the Gini index (rather than the entire cohort).

By contrast, models developed from regression methods are less easily understood. The beta coefficients from regression analysis are converted to an equation for formulation of clinical prediction rule. However, these clinical prediction rules assume that all of these beta coefficients are required at once to formulate a clinical decision rule. Due to the sequential nature of clinical reasoning and patient variables, some beta coefficients will be used, and some will be left out. This would make some of the elements of the clinical prediction rule from regression analysis redundant. By contrast, decision tree method generates a logical flowchart diagram that resembles a tree (9). This tree-like diagram with repeated partitioning of the original data into smaller groups (nodes) on a yes or no basis mimics clinical pathway reasoning. Furthermore, there is no requirement for clinicians to remember the individual score attributed to the variables. A potential disadvantage of the splitting rule used here is that it may be biased toward attributes that contain many different levels or scales. This may explain the choice of infarct volume, age, and GCS as the root node (at the first split).

The idea behind prediction is that the outcome can be “foretold” early. In this exploratory analysis, we have used the phrase “early associates of outcome” because of the use of MRI scans done at different time periods. In line with previous description, there was a steady improvement in prediction of coma outcome using clinical data only (5). Due to the life and death nature of this decision, the auROC should be above 0.90 and corresponded to outstanding discrimination (12) before being accepted. Observe that the GCS was not a helpful predictor at day 0 because these patients were intubated and admitted to intensive care. This meant that the GCS would be low. The usefulness of the GCS at day 2 indicated that if the patients have not regained consciousness it portends a poor outcome. This prediction reached a maximum at day 7 with auROC classified as outstanding. However, 7 days may be too long for the family to wait. This finding is consistent our earlier meta-analysis of the importance of clinical examination in the prognostication of anoxic coma outcome (5).

The finding from our exploratory analysis on the role of MR imaging is encouraging. We demonstrated that between day 0 and day 2, MR imaging measurement of infarct volume provided additional information for prognostication. This finding is consistent with the other observations on the use of MR imaging (13). We should acknowledge that our analyses were performed with the major assumption that the MR imaging findings do not significantly change between day 0 and day 7. There is a logistic difficulty of arranging multiple medical and nursing staff to ensure patient safety during MR scanning and as such we would not be able to perform MR imaging three times within 7 days. MR imaging data from neuroprotection trials in ischemic stroke showed a small increase between 12 h after onset and at 24 h (14). In this study, infarct expansion declined in the subacute period (14). With regards to the effect of the timing of MR imaging on the outcome, investigators have described a small decrease in infarct volume at 3 months when compared with the same infarct that was scanned within 7 days of stroke (15). However, these changes are less likely to affect the small infarct volume of 6 ml in our study.

Our approach of using the combined infarct volume in the prediction of outcome is different from other published studies on MR imaging (7, 13). In that study, the investigators had used different thresholds of ADC values to separate the outcomes among three different groups (13). Importantly the group who were alive in that study had infarct volume (ADC < 650 × 10^−6^ mm^2^/s) of less than 10% of total brain voxels. Other groups have found differences in regional ADC values across the whole brain between those with good and poor outcome (7, 16). However, the use of whole brain median ADC values to predict poor outcome is less easy to perform at the bedside compared with estimation of volume of 6 ml (7).

### Limitations

The limitations of this study have been described in a related paper on topography of hypoxic ischemic injury (10). These limitations include retrospective nature, small sample size (only 13% of the comatosed patient had MR scans), and potential for underestimation of white matter ischemic injury when using conventional imaging. Specific to this analysis, the small sample size limited our ability to perform a validation study using the method of partitioning the cohort into training and validation cohorts. The median time to MR imaging was 7 days in our study, and this raised the issue of infarct underestimation on the DWI images from the effect of pseudonormalization, which could have affected the appearance of ischemic injury in patients scanned between around day 4 and 10 (17, 18). This phenomenon affects the DWI sequences but not the FLAIR images and hence our choice to define the final ischemic injury by the union of the lesions on ADC, DWI and FLAIR images. Our paper may be criticized in the use of delayed scans to predict outcome at 1–2 days. As stated in the development of the model in Section “Materials and Methods,” we had assumed that the ischemic volume would remain constant (taking into account the DWI, ADC, and FLAIR ischemic volume). There are strong assumptions made in this study about the lack of changes in MR imaging findings as time elapsed from the initial ischemic event. These assumptions would require validation in prospective study. Although the median time to death after MR imaging was 6 days, there was the possibility of a self-fulfilling prophecy. The self-fulfilling prophecy as it relates to this retrospective study, opens up the possibility that the MRI findings may have influenced the treating physicians in the decision to continue or withdraw ongoing supportive treatment. Therefore, it is possible that supportive care could have been terminated in patients with large infarct volumes leading to death and strengthening the association.

### Conclusion

Our findings provide proof of concept for further exploration of the role of MR imaging in the early prognostication of hypoxic ischemic brain injury outcome. Decision tree analysis may be used to help with developing pathway for classifying coma outcome.

## Ethics Statement

The study was approved by the Ethics Committee. A waiver of consent was given due to the retrospective and observational nature of this study.

## Author Contributions

Study concept and design: SS and TP. Acquisition of clinical data and MRI rating: SS. Drafting of manuscript: SS, JL, HM, BC, RB, and TP. Critical revision of manuscript for intellectual content: all the authors. Statistical analysis: JC, RB, and TP.

## Conflict of Interest Statement

The authors declare that the research was conducted in the absence of any commercial or financial relationships that could be construed as a potential conflict of interest.
